# Radiotherapy combined with anti-CEACAM1 immunotherapy to induce survival advantage in glioma

**DOI:** 10.1007/s12672-023-00638-x

**Published:** 2023-03-16

**Authors:** Jinhu Li, Yi Chen, Yimin Fan, Hongqin Wang, Wei Mu, Xiaodong Liu

**Affiliations:** 1 Department of Neurosurgery, The First Hospital of Shanxi Medical University, 85 Jiefang South Road, Taiyuan, Shanxi China; 2 Department of Interventional Radiology, Shanxi Provincial People’s Hospital, Taiyuan, China

**Keywords:** Glioma, Immunotherapy, Radiotherapy, Immune checkpoint, Immune escape

## Abstract

**Background:**

We aimed to observe the effect of radiotherapy on the expression of immune checkpoint molecule CEACAM1 in patients with glioma and the therapeutical effect of radiotherapy combined with blockade of CEACAM1 in mice with intracranial gliomas.

**Methods:**

The expression of CEACAM1 on T-lymphocytes in the peripheral blood of patients with glioma was detected before and after radiotherapy; GL261 murine glioma cells (stably transfected with the luciferase gene) were implanted in the right caudate nucleus of C57BL/6 mice, and tumour growth was observed using the small animal in vivo imaging system. Mice were divided into 4 groups: (1) the isotype control; (2) the radiotherapy; (3) the anti-CEACAM1 treatment; and (4) the combination therapy. The survival of mice after treatment was recorded; the expression of CEACAM1 on murine glioma cells was detected by immunohistochemistry before and after radiotherapy; flow cytometry was adopted to detect CD8^+^ T-cells (Treg) (CD4^+^FoxP3^+^CD25^+^) among mouse brain-infiltrating T-cells; serum levels of IFN-γ and IL-10 were detected by ELISA; proliferation and apoptosis were observed by immunohistochemistry; Retrospective RNA-seq data analysis was conducted in a cohort of 325 patients with glioma in the Chinese Glioma Genome Atlas (CGGA) database and 702 patients in The Cancer Genome Atlas (TCGA) database.

**Results:**

The expression of CEACAM1 on CD4^+^ and CD8^+^ T-cells in the peripheral blood of patients with glioma was significantly higher 1 week after radiotherapy than before radiotherapy and was further increased 1 month after radiotherapy. Combined therapy notably inhibited the growth of intracranial tumours in mice and prolonged their survival time, with some mice being capable of surviving long-term (> 90 d). Immunohistochemistry revealed that the expression of CEACAM1 in murine glioma tissues after radiotherapy was elevated in a time-dependent manner. Flow cytometry analysis showed an increase in mouse brain-infiltrating CD8^+^ T-lymphocytes, a decrease in Treg cells, and an increase in CD8^+^ T/Treg cells after treatment. ELISA demonstrated the elevated levels of IFN and decreased levels of IL-10 in the serum of mice in the combination therapy group.

**Conclusions:**

Radiotherapy combined with CEACAM1 inhibitors resulted in strong and durable anti-tumour immune responses against murine glioma and long-term survival of some mice. Hence, this study is expected to offer new effective immunotherapy strategies against glioma.

## Background

Gliomas are the most common malignancies of the central nervous system [1]. Glioblastoma (WHO grade IV) accounts for approximately 55% of glioma, the remaining 45% of glial tumor are composed of several different histologies including diffuse astrocytoma (WHO grade II), anaplastic astrocytoma (WHO grade III) and oligodendroglioma (WHO grade II and III) [2]. The prognosis of patients is poor despite aggressive comprehensive therapy, with a median survival time of 14.6 months and a 3-year survival rate of only 10% for those with glioblastoma [1]. Median survival is longer in WHO grade II-III glioma compared with glioblastoma as survival times of up to 10 years [2]. Although immunotherapy has been successfully used against a variety of malignant tumours, its efficacy in gliomas remains poor, mainly because gliomas promote the formation of an immunosuppressive tumour microenvironment that greatly inhibits anti-tumour immune responses [3]. Previous studies have shown that radiotherapy increased the immunogenicity of tumours by killing tumour cells and leading to the simultaneous release of neo-antigens. Meanwhile, radiotherapy was found to induce the expression of new immune checkpoint molecules that can be blocked by antibodies to enhance the immune response of effector T-cells [4]. In addition, radiotherapy might target a series of immune activations stimulated by immunotherapeutic agents at tumour antigens instead of normal tissue auto-antigens, and hence reduce the occurrence of immune-related adverse events [5]. Preliminary studies showed the abnormal expression of carcinoembryonic antigen cell adhesion molecule 1 (CEACAM1) in glioma tissues, which was closely associated with multiple clinicopathological factors of patients with glioma [6]. This study aimed to investigate the expression of CEACAM1 on CD4^+^ and CD8^+^ T-cells in the peripheral blood of patients with glioma before and after radiotherapy. In addition, we observed the efficacy of radiotherapy combined with immune checkpoint CEACAM1 inhibitors on intracranial glioma in murine models and its impact on anti-tumour immune responses.

## Material and methods

In this study, we first detected the expression of CEACAM1 on T lymphocytes in peripheral blood of patients with glioma before and after radiotherapy. Subsequently, the murine intracranial gliomas models were constructed, and the mice were treated with radiotherapy combined with monoclonal antibodies blocking CEACAM1 and the survival was observed. In addition, the expression of CEACAM1 in brain gliomas were detected by immunohistochemistry before and after radiotherapy, and the expression of CD4^+^, CD8^+^ T-cells and Tregs in brain infiltrating lymphocytes (BILs) were observed with flow cytometry. IFN-γ and IL-10 in the peripheral blood of mice were determined by ELISA.

### Clinical data of patients with glioma

We collected the clinical data of patients with primary glioma undergoing surgical treatment at the Neurosurgery Department of the First Hospital of Shanxi Medical University and subsequent radiotherapy within 1 month at the Radiotherapy Department from March to December 2019. Are inclusion criteria included patients aged 18–80 years who were pathologically diagnosed with glioma, had complete clinical data, and were expected to undergo radiotherapy after glioma resection surgery. Whereas our exclusion criteria included patients who had received immunosuppressants in the previous 3 months and presented severe infections, coagulation disorders, immune-related diseases, and tumours in other systems.

Based on these criteria, a total of 20 patients with glioma were enrolled in the study, consisting of 8 men and 12 women; patients ranged in age from 33 to 79 years with a mean of 61 years, including 3 patients with WHO grade II glioma, 8 patients with WHO grade III glioma, and 9 patients with WHO grade IV glioma (Table 1). Fresh peripheral blood (5-10 ml) was collected from patients early in the morning before radiotherapy, at 1 week after radiotherapy, and at 1 month after radiotherapy (routine radiation dose: 1.8–2.0 Gy per fraction, 30 fractions), followed by flow cytometry testing within 3 h.

**Table 1:** Demographic information

Gender	No. of patients
Male	8
Female	12
Age (Mean, Range)	61 (33–79)
≥ 60	11
< 60	9
WHO	
II	3
III	8
IV	9

### Antibodies

Therapeutic antibodies were as follows: anti-CEACAM1 mAb (Clone MAb-CC1; Cat. #: 134504; Biolegend, Santa Cruz, CA, USA) and Mouse IgG1 isotype control (Clone MOPC-21; Cat. #: 400153; Biolegend). Antibodies for flow cytometry and immunohistochemistry were as follows: anti-mouse CD3e (Clone 145-2C11; Cat. #: 15–0031-82; eBioscience, San Diego, CA, USA), anti-mouse CD4 (Clone GK1.5; Cat. #: 12–0041-82; eBioscience), anti-mouse CD8a (Clone 53–6.7; Cat. #: 11–0081-82; eBioscience), anti-mouse CD25 (Clone PC61.5; Cat. #: 25–0251-82; eBioscience), anti-mouse Foxp3 (Clone FJK-16 s; Cat. #: 11–5773-82; eBioscience), anti-mouse CEACAM1 (Clone 723629; Cat. #: MA5-24338; eBioscience), anti- human CD3 (Clone OKT3; Cat. #: 14–0037-82; eBioscience), anti- human CD4 (Clone RPA-T4; Cat. #: 11–0049-42; eBioscience), anti-mouse CD8a (Clone RPA-T8; Cat. #: 12–0088-42; eBioscience), anti-human CEACAM1 (Clone YTH71.3; Cat. #: MA5-17003; eBioscience),anti-mouse ki-67(SAB5700770; Sigma-Aldrich) and In Situ Cell Death Detection Kit (11684817910;Roche).

### Flow cytometry

Monoclonal antibodies were added to the peripheral blood samples collected from patients with glioma and incubated at 37 °C for 30 min. Hemolysin (Boster, Wuhan, Hubei, China) was then added to samples before centrifugation (800 × *g*, 5 min), washing, re-suspension, and analysis using a BD FACSCanto™ II flow cytometer (BD Biosciences, Franklin Lakes, NJ, USA). After C57BL/6 mice were euthanised, their brain tissues were cut into small pieces, ground-up, filtered, and re-suspended before the addition of lymphocyte separation medium (Tianjin Haoyang Biological Products Technology Co., Ltd., Tianjin, China). Subsequently, each mixture was centrifuged (800 × *g*, 20 min) to collect the middle white cell layer, followed by washing and re-suspension with PBS (Boster, Wuhan, Hubei, China). Monoclonal antibodies were then added and samples were incubated at 37 °C for 30 min in the dark, followed by washing and re-suspension in PBS before analysis. All data were analysed using the FlowJo software (FlowJo, LLC, Ashland, OR, USA).

### Construction of animal model and grouping

For the generation of the animal model, 6- to 9-week-old female C57BL/6 mice (purchased from the Shanxi Medical University Animal Center) were anaesthetised. The head of each mouse was stabilized and punctured at 1 mm behind the anterior fontanelle and at 2 mm right of the midline. Then, a 10 μL suspension of GL-261 murine glioma cells (GL-261-Luc) that had been transfected with the luciferase gene (5 × 10^5^ cells/mL) were stereotactically injected into the mouse brain and the syringe was left in place for 3 min before being withdrawn. Bone wax was then used to seal the burr holes, and the incision was sutured and disinfected. The volume of the intracranial tumour of mice was detected and photographed using an in vivo imaging system (BRUKE, Billerica, MA, USA). Mice were randomly divided into 4 groups (5 mice per group): (1) the isotype control group (isotype control antibodies); (2) the radiotherapy group (radiotherapy as monotherapy); (3) the anti-CEACAM1 treatment group (anti-CEACAM1 monoclonal antibody); and (4) the combination therapy group (radiotherapy + anti-CEACAM1 monoclonal antibody). Mice in the (3) and (4) groups were intraperitoneally injected with anti-CEACAM1 monoclonal antibodies (250 μg) at 12 d after tumour implantation, whereas mice in the (1) group were intraperitoneally injected with isotype control monoclonal antibodies. The isodose distribution of irradiation on mice was evaluated, with the irradiation field covering all brain tissues. 6 MV X-ray, absorbed dose rate: 2.0 Gy/min, source to skin distance: 100 cm, irradiation field size: 20 cm × 2 cm, machine head 180°. Tissue equivalent materials (1 cm) were placed below the head of each mouse. At 12 d after tumour implantation, mice in the (2) and (4) groups were irradiated to the head with a 10 Gy single fraction using a linear accelerator (Varian, Palo Alto, CA, USA). The health of the nervous system of mice, toxicity, and side-effects were regularly evaluated, such as dietary status, activity and posture, body weight, and hair. Mice in each group were subdivided into 2 groups: mice in the first subgroup were used for determining the survival time and plot the survival curve; mice in the other subgroup were killed at a given time after treatment (1 week or 1 month after treatment) to collect brain tissue and peripheral blood samples for subsequent experiments.

### Haematoxylin and eosin staining and immunohistochemistry testing

Specimens of murine intracranial tumour tissues were formalin-fixed and paraffin-embedded. HE staining and immunohistochemistry were performed following routine procedures. The Aperio Digital Pathology Slide Scanner (Aperio Technologies, Vista, CA, USA) was used to capture images. The immunoreactive scoring (IRS) was adopted to evaluate the level of expression of CEACAM1 based on the staining intensity and scope, as follows: IRS = percentage of positive cells × staining intensity. Percentage of positive cells: 0% was assigned a score of 0; 0–25% was assigned a score of 1; 25–50% was assigned a score of 2; 50–75% was assigned a score of 3; 75–100% was assigned a score of 4. Staining intensity: absent staining = 0; weak staining = 1; moderate staining = 2; strong staining = 3.

### Expression of IFN-γ and IL-10 in the peripheral blood of mice

Peripheral blood samples were obtained from mice to collect the supernatant. The concentrations of interferon (IFN)-γ (pg/mL) and IL-10 (pg/mL) were identified using ELISA kits (Cat. #: ab282874 and ab203359, respectively, Abcam, Cambridge, UK), according to the manufacturer’s instructions.

### Bioinformatics analysis

#### Public database

The patient transcriptome data and corresponding clinical information used in this study were derived from the Chinese Glioma Patient Genome Atlas (CGGA, http://www.cgga.org.cn/) and The Cancer Genome Atlas (TCGA, https://www.cancer.gov/about-nci/organization/ccg/research/structural-genomics/tcga) public database.

#### CEACAM1 expression in Glioma

Use R software package “ggplot2” and “pheatmap” to plot or visualize the date of Clinicopathologic features of glioma associated with CEACAM1 gene and expression of CEACAM1 gene in MGMT, IDH, 1p19q and grade.

#### Functional enrichment analysis

The most relevant genes of CEACAM1, or a characteristic gene list of the cell cluster, were uploaded to the Database for Annotation, Visualization, and Integrated Discovery (DAVID, v6.8). The official gene symbol was selected as an identifier, and Homo sapiens was selected as species. Finally, Gene Ontology (GO) analysis and Kyoto Encyclopedia of Genes and Genomes (KEGG) pathway analysis enrichment results were obtained. The top six results in ascending order of P-­value (P < 0.05) were displayed in this study.

#### Association between CEACAM1 expression and Glioma survival prognosis

Statistical analyses and visualization were performed in R (ver-sion 4.2.2) and IBM SPSS Statistics (version 26.0). The Kaplan–Meier (KM) survival curve analysis is implemented by R software package “Survival”and “Survminer”. Cox regression or logrank test was used to analyze the relationship between CEACAM1 expression and the survival rate of patients with glioma.

#### Association between CEACAM1 expression and Radio in Glioma survival prognosis

The Kaplan–Meier (KM) survival curve analysis is implemented by R software package “Survival” and “Survminer”. Cox regression or logrank test was used to analyze the relationship between CEACAM1 expression and the survival rate of Radio in patients with glioma.

### Statistical analysis

Statistical analysis was performed using GraphPad Prism version 8.0 (GraphPad Software Inc., San Diego, CA, USA) and SPSS (version 19.0; IBM Corp, Armonk, NY, USA). Continuous variables subject to normal distribution were denoted by the mean ± standard deviation. Comparisons of multiple groups at the same time points were conducted using one-way ANOVA, comparisons at different time points were conducted using repeated ANOVA, and further pairwise inter-group comparisons were conducted using the least significance difference test. Continuous variables that did not obey normal distribution (e.g., the expression level of CEACAM1 on the surface of CD4^+^ T lymphocytes in the peripheral blood of patients) were expressed as *M(Q1,Q3)*. Data analysis of multiple relevant samples at different time points was conducted using the Friedman *M* test for the comparison, and further pair comparison was conducted by *q* test. Survival analysis was conducted using the Kaplan–Meier method, and the comparisons of multiple groups were conducted using a log-rank test. *P* < 0.05 indicated statistical significance.

## Results

### Expression of CEACAM1 on T-cells in the peripheral blood of patients with glioma before and after radiotherapy

**Fig. 1:**
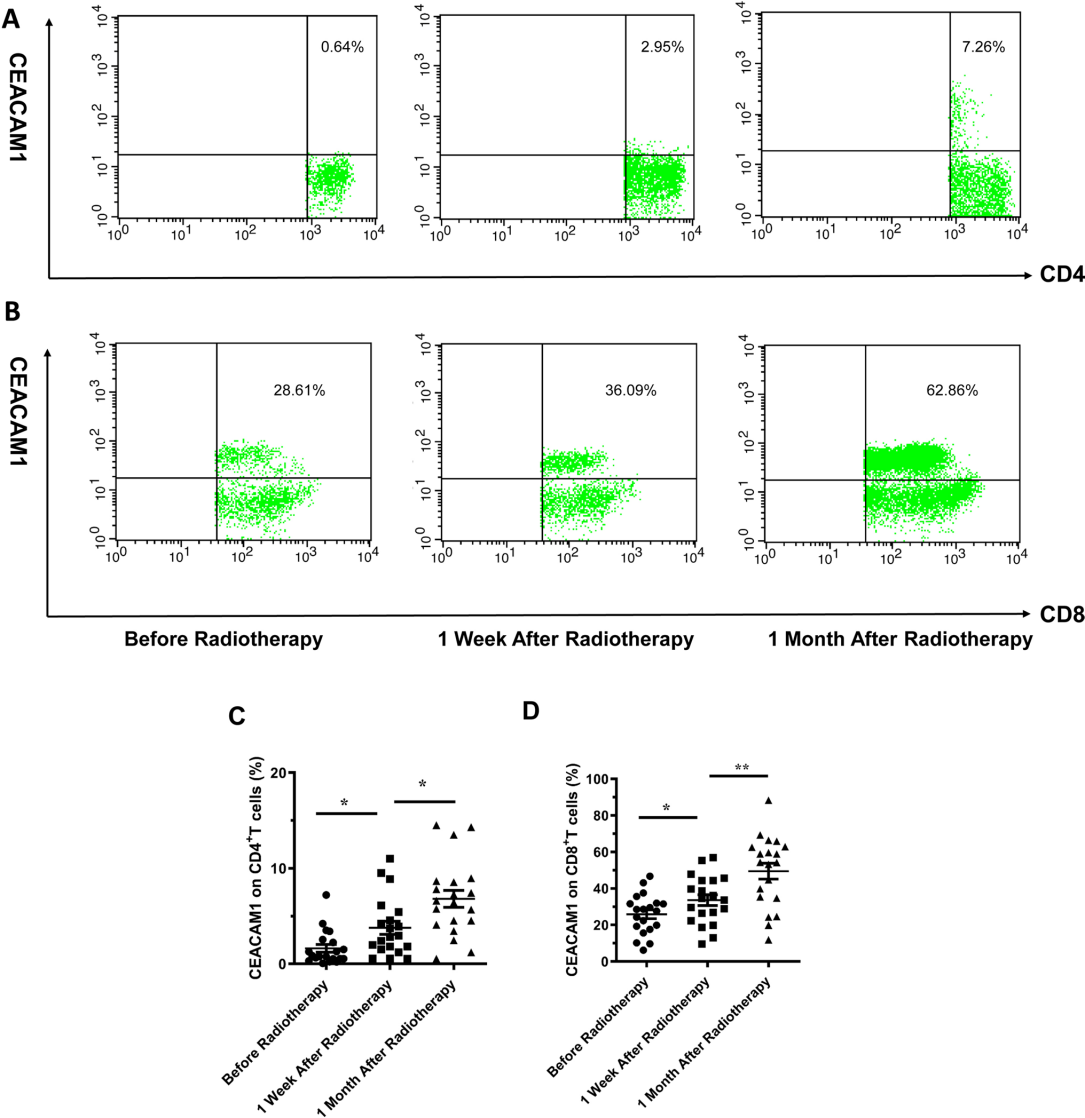
Expression of CEACAM1 on T-lymphocytes in the peripheral blood of postoperative patients with glioma before and after radiotherapy. **A**, **B** Flow-cytometric graphs of the expression of CEACAM1 on CD4^+^ and CD8^+^ T-lymphocytes in the peripheral blood of patients with postoperative glioma before and after radiotherapy. **C** Statistical graph of the percentage of CEACAM1^+^ CD4^+^ T-cells. **D** Statistical graph of the percentage of CEACAM1^+^ CD8^+^ T-cells. *: *P* < 0.05, **: *P* < 0.01

We adopted flow cytometry to detect the expression of CEACAM1 on T-cells in the peripheral blood of postoperative patients before radiotherapy, 1 week after radiotherapy, and 1 month after radiotherapy (Fig. 1A, B), and observed that the expression levels of CEACAM1 on CD4^+^ T-cells (*M(Q1,Q3)*) in the three groups were 0.82 (0.53,2.47), 2.88 (1.58,5.21), and 6.52 (4.21,8.63), respectively (Fig. 1C); the differences between the three groups were statistically significant (*P* < 0.001). For CD8^+^ T-cells, the expression levels of CEACAM1 in the three groups were 25.88 ± 10.89, 33.61 ± 13.26, and 49.48 ± 19.49, respectively (Fig. 1D); with significant differences between the groups (*P* < 0.001). Compared with results recorded before radiotherapy, the expression level of CEACAM1 on CD4^+^ T-lymphocytes increased one week after radiotherapy (*P* = 0.0103) and increased further one month after radiotherapy (*P* = 0.0096). The expression level of CEACAM1 on CD8^+^ T-lymphocytes increased one week after radiotherapy, but the difference was not statistically significant (*P* > 0.05); the expression level, however, did significantly increase one month after radiotherapy (*P* = 0.0046).

### Intracranial glioma proliferation and murine survival

**Fig. 2:**
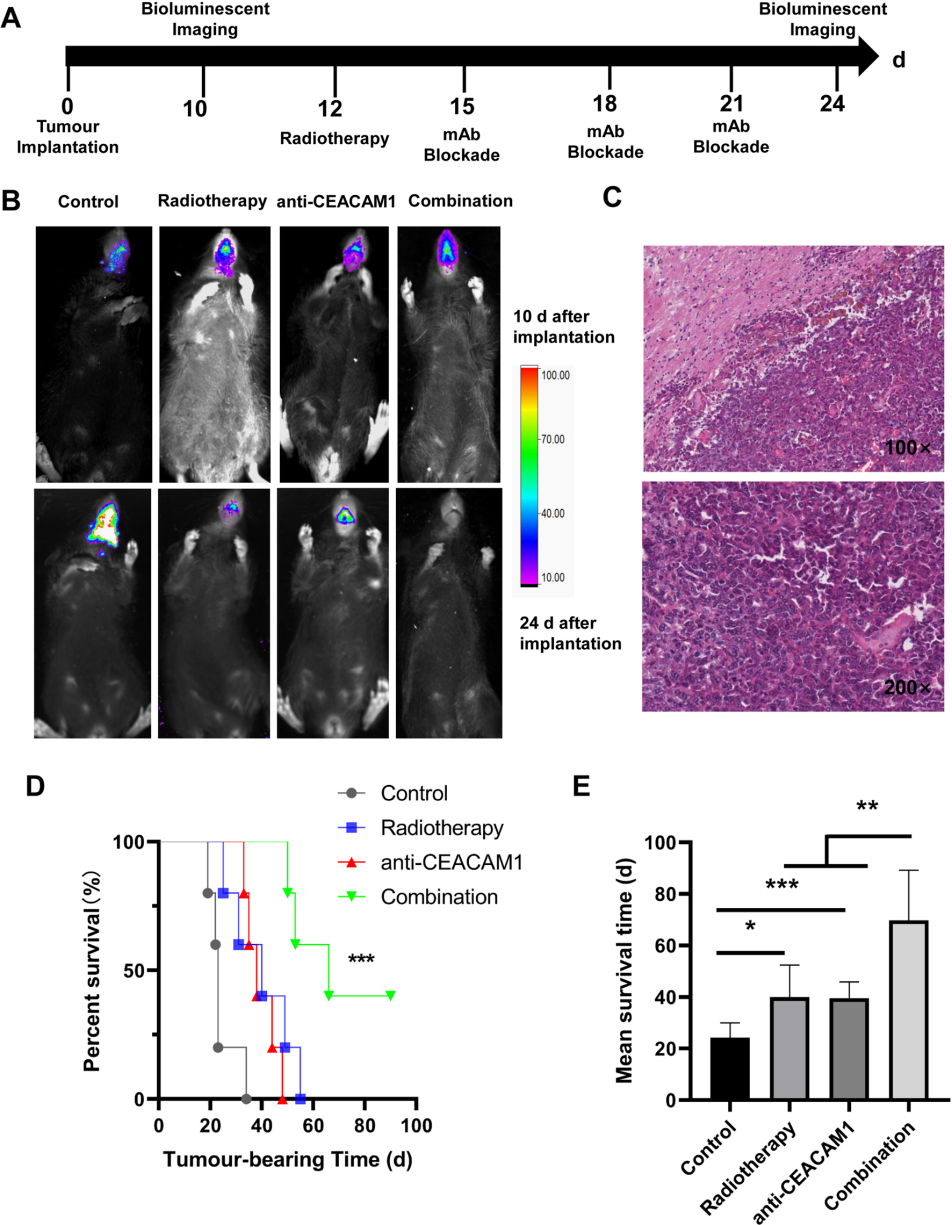
Growth of in situ gliomas and survival of mice in different treatment groups. **A** Study timeline. **B** Representative images at 10 and 24 d after tumour implantation in different groups; at 10 d, the number of photons for the 4 mice (from left to right) were: 386596 P/s, 914306 P/s, 454961 P/s, and 871112 P/s; at 24 d the numbers were: 4658120 P/s, 225346 P/s, 697543 P/s, and 0 P/s). **C** Images of HE-stained murine tumours. **D** Survival curves of mice in different groups. **E** Mean survival time of mice in different groups. *: *P* < 0.05, **: *P* < 0.01, ***: *P* < 0.001

We successfully constructed a C57BL/6-GL261-Luc mouse model of glioma in situ. We observed the orthotopic tumour engraftment by luciferase imaging on days 10 after tumour implantation. Mice in the radiotherapy group and combination therapy group were treated with radiotherapy on days 12. The anti-CEACAM1 mAbs were intraperitoneally injected (i.p.) on days 15, 18, and 21. The controls received isotype control monoclonal antibodies treatment. We then reassessed the tumor progression by luciferase imaging on day 24 (Fig. 2A). We found that murine tumour growth was inhibited in the radiotherapy group and the anti-CEACAM1 group compared with that in the isotype control group, whereas murine intracranial tumour volume shrank significantly in the combination therapy group with some mice experiencing even tumour dissipation (Fig. 2B). In addition, our staining analysis of HE showed that the tumour characteristics of glioma were consistent with its pathological features (Fig. 2C). The median survival time and 95% confidence interval of the isotype control group, the radiotherapy group, anti-CEACAM1 group, and combination therapy group were 23.00 (22.12, 23.88) d, 40.00 (20.68, 59.32) d, 38.00 (31.56, 44.44) d, and 66.00 (38.09, 93.9) d, respectively. The survival of the combination therapy group increased significantly, and some of the mice lived for more than 90 d (2/5); the difference was statistically significant between the four groups (*P* < 0.001), and compared with the isotype control group, the mean survival of the radiotherapy group (*P* = 0.013), anti-CEACAM1 group (*P* < 0.001) and combination therapy group (*P* < 0.001) were increased (Fig. 2D, E).

### Expression of CEACAM1 on murine in situ glioma cells before and after radiotherapy

**Fig. 3:**
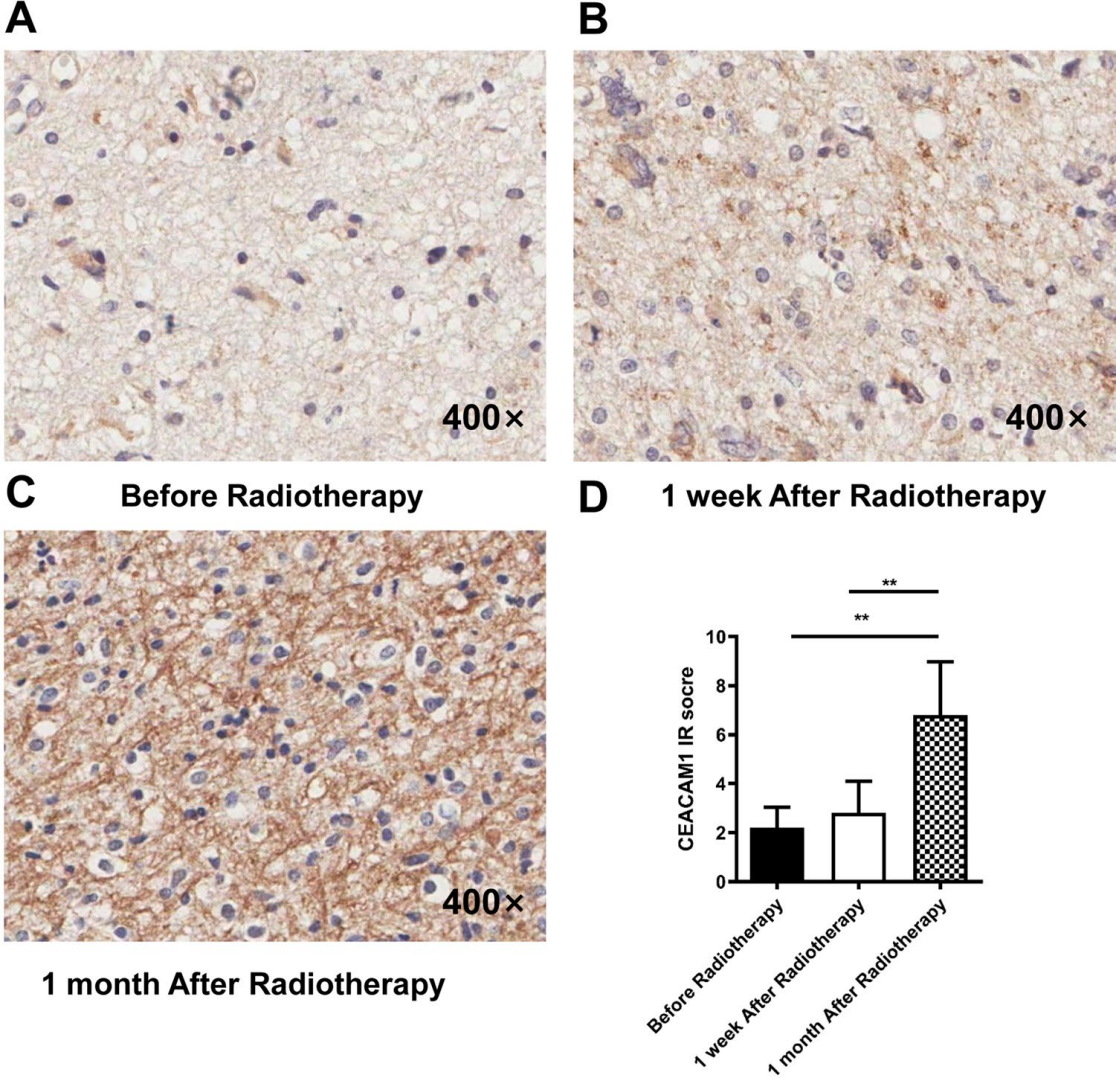
Expression of CEACAM1 in murine intracranial glioma cells before radiotherapy, 1 week and 1 month after radiotherapy as detected by immunohistochemistry. **A–C** Representative images of immunohistochemically stained murine glioma tissues at different time windows before and after radiotherapy. **D** Immunohistochemistry staining intensity scores. **: *P* < 0.01

We then adopted more intracranial mouse models of glioma. We selected mice before radiotherapy, 1 week after radiotherapy, and 1 month after radiotherapy and euthanised them. We then obtained the tumour tissues and performed immunohistochemistry to detect the expression of CEACAM1 on murine intracranial glioma cells (Fig. 3A–C). We found that the expression of CEACAM1 was mostly localized on the cell membrane and in some cases in the cytoplasm of glioma cells. We also noticed that at 1 week after radiotherapy, the expression of CEACAM1 was elevated; however, the observed differences were not statistically significant (*P* > 0.05). However, at 1 month after radiotherapy, the expression of CEACAM1 was significantly elevated (IR scores of the expression of CEACAM1: 2.20 ± 0.84, 2.80 ± 1.30, and 6.80 ± 2.17 respectively, *F* = 13.21, *P* = 0.0022) (Fig. 3D).

### Distribution of CD4^+^ T-cells, CD8.^+^ T–cells, and Treg cells in murine brain infiltrating lymphocytes (BILs)

**Fig. 4:**
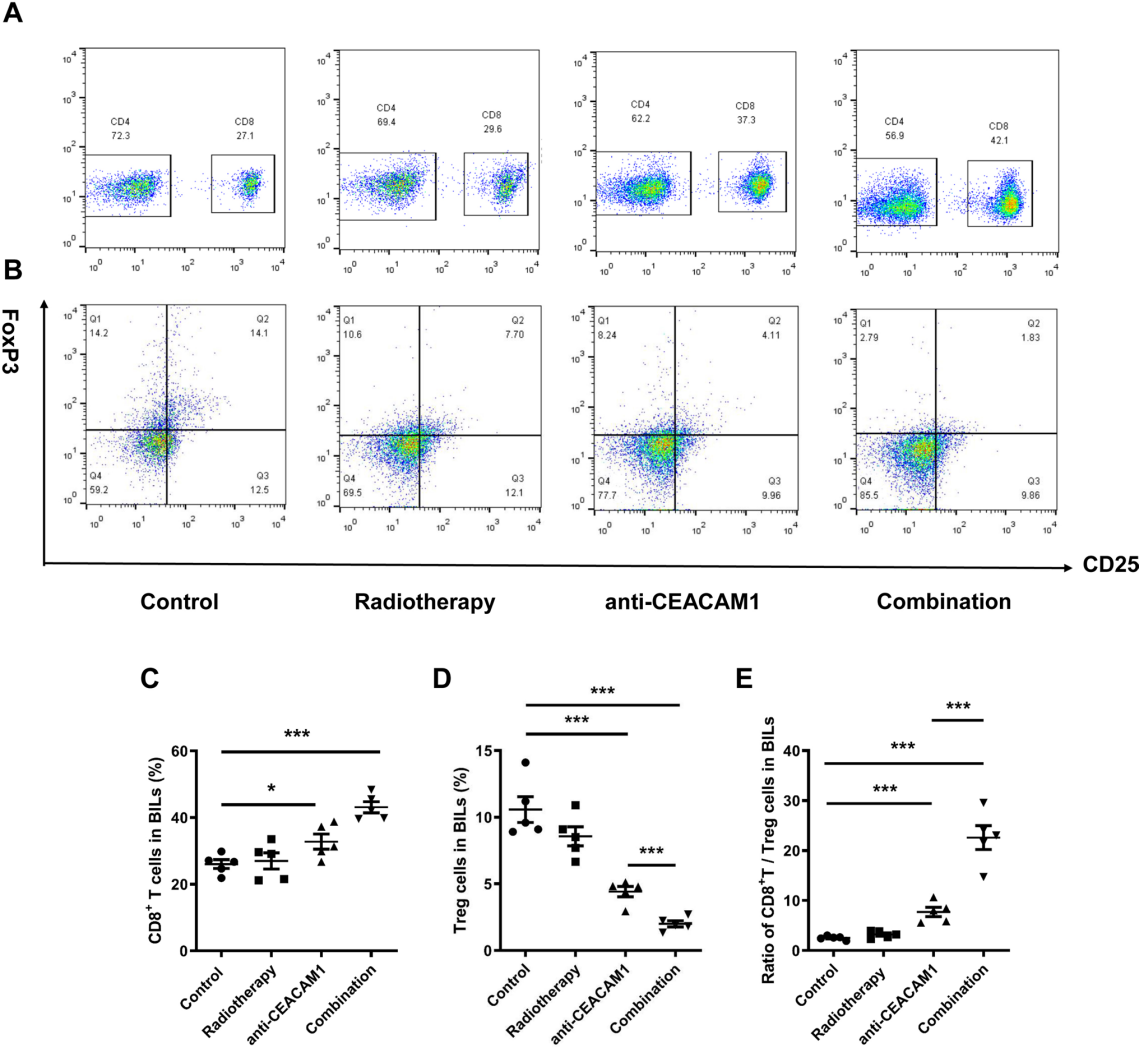
Percentage of CD8^+^ T-cells in BILs, percentage of Treg cells in BILs, and ratio of CD8/Treg detected by flow cytometry in mice of different groups. **A** and **B** Representative images of flow cytometry illustrating the percentage of CD8^+^ T-cells in BILs and the percentage of Treg cells in BILs in mice of different groups. **C** Statistical graph on the percentage of CD8.^+^ T-cells. **D** Statistical graph on the percentage of Treg cells. **E** Statistical graph on the ratio of CD8/Treg. *: *P* < 0.05, ***: *P* < 0.001

We performed flow cytometry to detect the percentage of CD8^+^ T-cells and Treg cells in murine BILs of different groups at 21 d after treatment and observe the ratio of CD8/Treg cells (Fig. 4 A and B). We observe that the percentage of CD8^+^ T-cells in mice of the radiotherapy group did not change significantly. However, the percentage of CD8^+^ T-cells was increased in mice of the anti-CEACAM1 and combination therapy groups, with the latter being even greater (CD8/CD3, *P* = 0.033, *P* < 0.001, respectively) (Fig. 4C). Meanwhile, we found that the percentage of Treg cells in mice of the anti-CEACAM1 treatment group was decreased, with that of Treg cells in the combination therapy group being even greater (*P* < 0.001). In addition, the percentage of Treg cells in the combination therapy group was lower than that in the anti-CEACAM1 treatment group (*P* < 0.001) (Fig. 4D). Further studies showed that the differences in the ratio of CD8/Treg between the isotype control and radiotherapy groups were not statistically significant (*P* > 0.05). We specifically noticed that the ratio of CD8^+^/Treg in the anti-CEACAM1 treatment group was elevated, whereas that in the combination therapy group was elevated even more (*P* < 0.001). Interestingly, we detected that the ratio of CD8^+^/Treg in the combination therapy group was lower than that in the anti-CEACAM1 treatment group (*P* < 0.001) (Fig. 4E).

### Expression of IFN-γ and IL-10 cytokines in the peripheral blood detected by ELISA

**Fig. 5:**
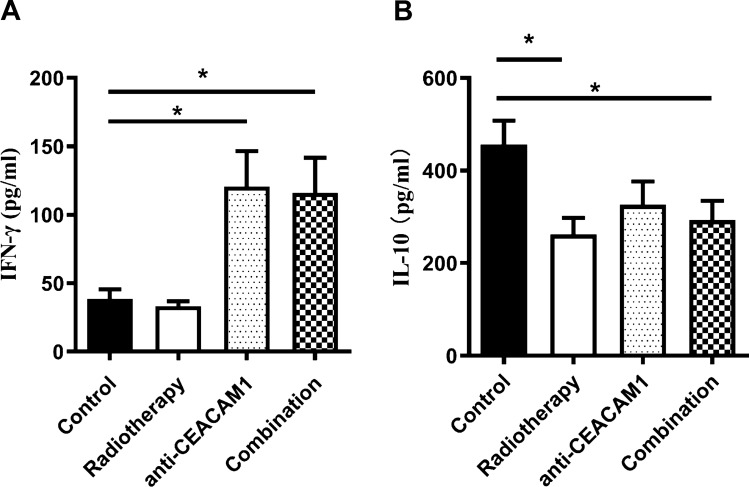
Expression of IFN-γ and IL-10 in the peripheral blood of mice in different groups detected by ELISA. **A** Expression of IFN-γ in the peripheral blood of mice in different groups. **B** Expression of IL-10 in the peripheral blood of mice in different groups. *: *P* < 0.05

We found that the radiotherapy group exhibited a lower level of expression of IFN-γ compared with that in the isotype control group; however, these differences were not statistically significant (*P* > 0.05). We noticed that the level of expression of IFN-γ in the anti-CEACAM1 treatment and combination therapy groups was elevated (*P* = 0.0152, *P* = 0.0197, respectively); however, the inter-group differences were not statistically significant (*P* > 0.05) (Fig. 5A). We also detected that the level of expression of IL-10 was lower in the radiotherapy group (*P* = 0.0144), and the combination therapy group (*P* = 0.0382), compared with that in the isotype control group, but differences with the anti-CEACAM1 treatment group were not statistically significant (*P* > 0.05) (Fig. 5 B).

### The proliferation and apoptosis of glioma cells were observed by immunohistochemistry

**Fig. 6:**
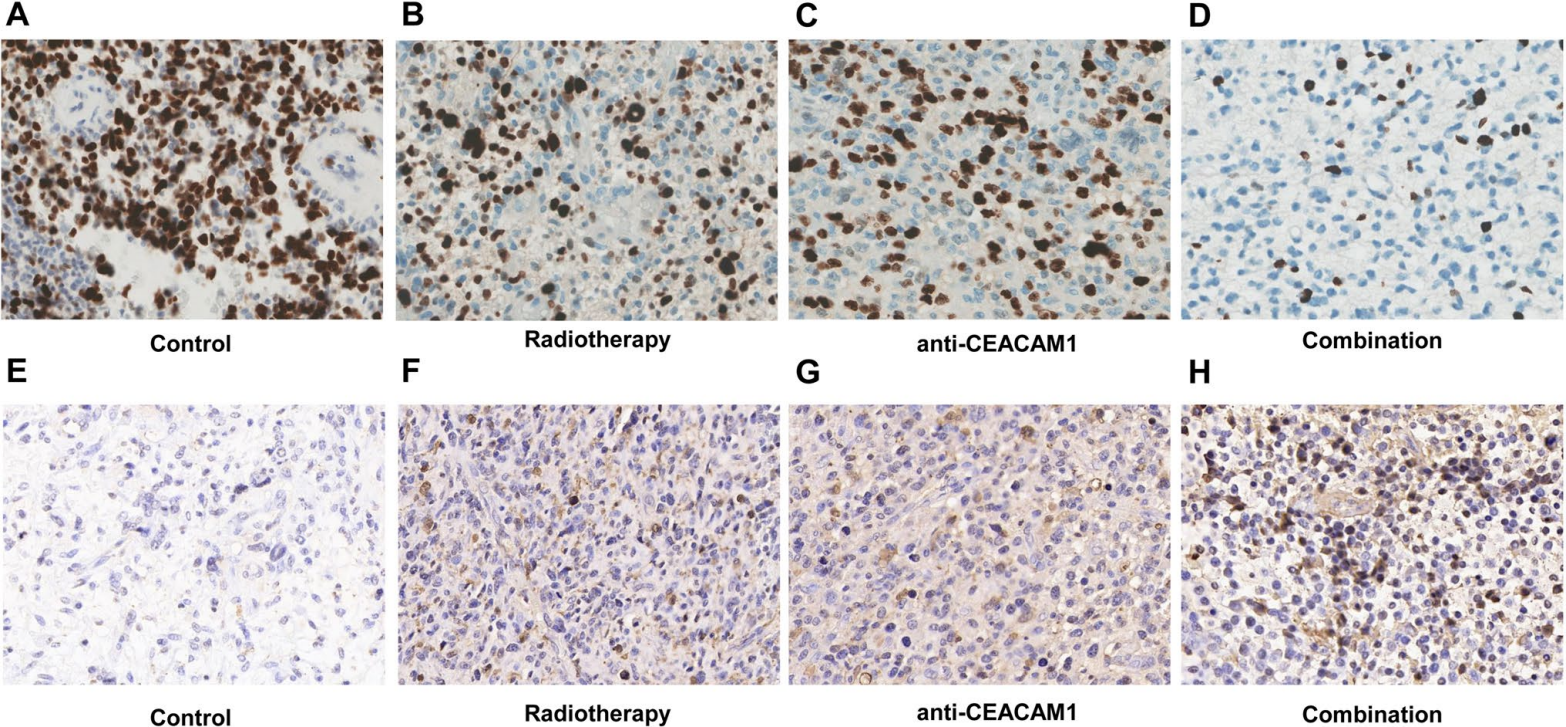
Ki-67 and TUNEL for proliferation and apoptosis in the tumor tissues in different groups detected by immunohistochemistry.** A**–**D** Expression of Ki-67 in the glioma tissues of mice in different groups. **E**–**H** The apoptosis of tumor tissues in different groups

We performed immunohistochemistry to observe the proliferation (ki-67) and apoptosis (tunel) of glioma cells. We found that the radiotherapy group exhibited a higher level of expression of ki-67 compared with that in other groups, and lowest in the combination therapy group (Fig. 6A–D). In addition, we observed the apoptosis of tumor in each group, and the results showed that The apoptosis rate of glioma cells in the combined treatment group was significantly higher than that in the other groups (Fig. 6E–H).

### Expression of CEACAM1 is enriched in gliomas with higher malignancy

**Fig. 7:**
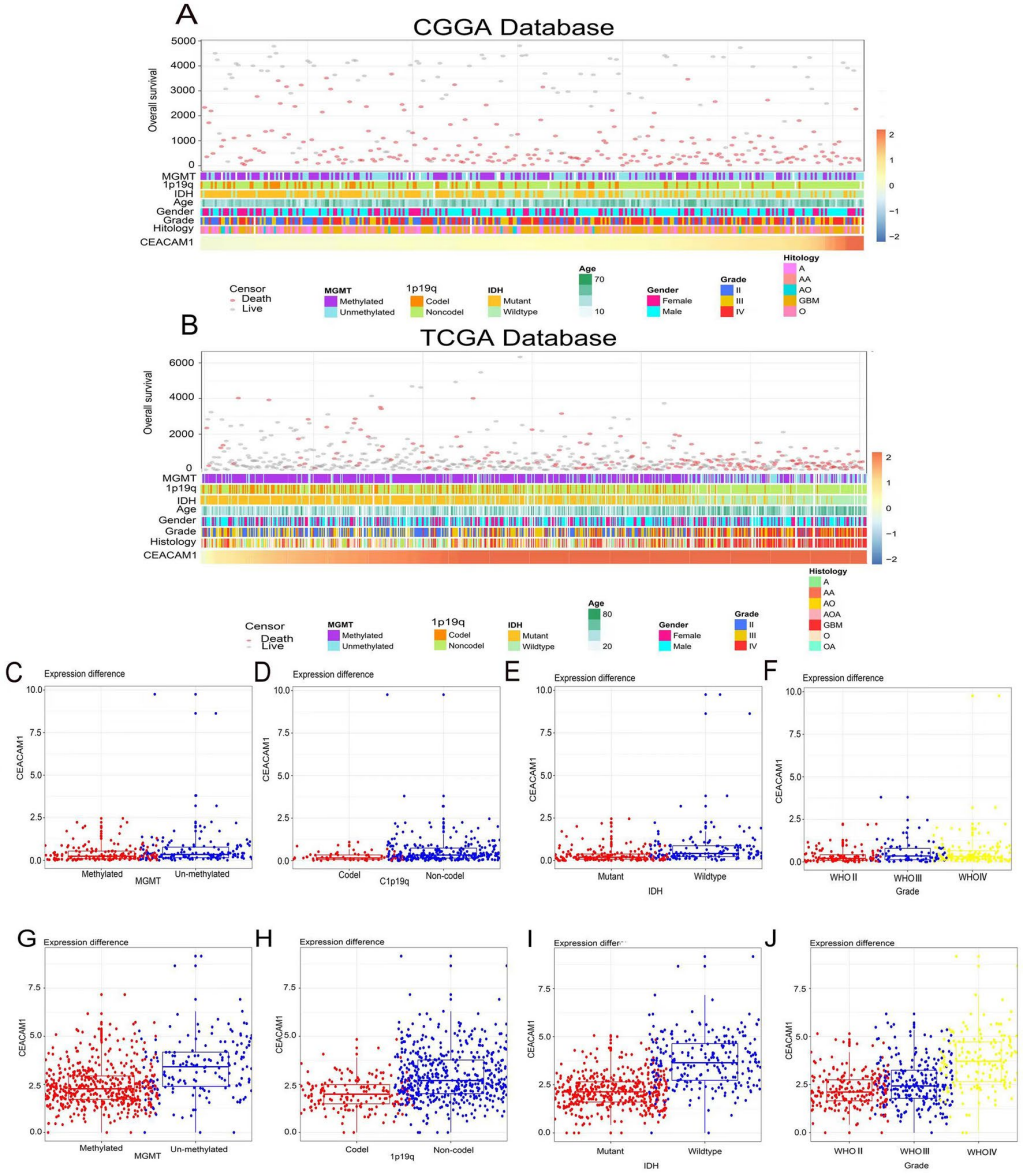
Association between CEACAM1 and clinicopathological characteristics of gliomas. **A** The landscape of CEACAM1-related clinicopathological features of gliomas in the Chinese Glioma Genome Atlas (CGGA) database. **B** The landscape of CEACAM1-­relatedclinicopathological features of gliomas in the The Cancer Genome Atlas (TCGA) database. **C**–**F** Distribution of glioma-related gene CEACAM1 in MGMT, IDH, Grade and 1p19q. **G–J** Distribution of glioma-related gene CEACAM1 in MGMT, IDH, Grade and 1p19q

Patients with varying expression levels of CEACAM1 showed distinct patterns of clinical and pathological characteristics. Increases in CEACAM1, MGMT promoter methylation status, 1p/19q codeletion status, IDH mutation status, WHO grade, and histology diagnosis showed asymmetric distributions in the CGGA and TCGA datasets (Fig. 7A, B). Comparative analysis was conducted with different groups of these samples. In the CGGA database, samples without 1p/19q codele- tion showed a higher expression of CEACAM1 (Fig. 7D). Moreover, CEACAM1 was highly enriched in IDH-wildtype gliomas (Fig. 7E) and higher-­grade gliomas (Fig. 7F). The above results were validated in the TCGA database (Fig.7H–J). CEACAM1 was highly expressed in samples without MGMT promoter meth-ylation in the TCGA database (Fig. 7H). The expression of CEACAM1 had the same trend in the CGGA database, although this difference was not statistically significant (Fig. 7C). Overall, these results show that gliomas with higher malignancy are enriched for CEACAM1.

### CEACAM1 regulates the inflammatory response in gliomas

**Fig. 8:**
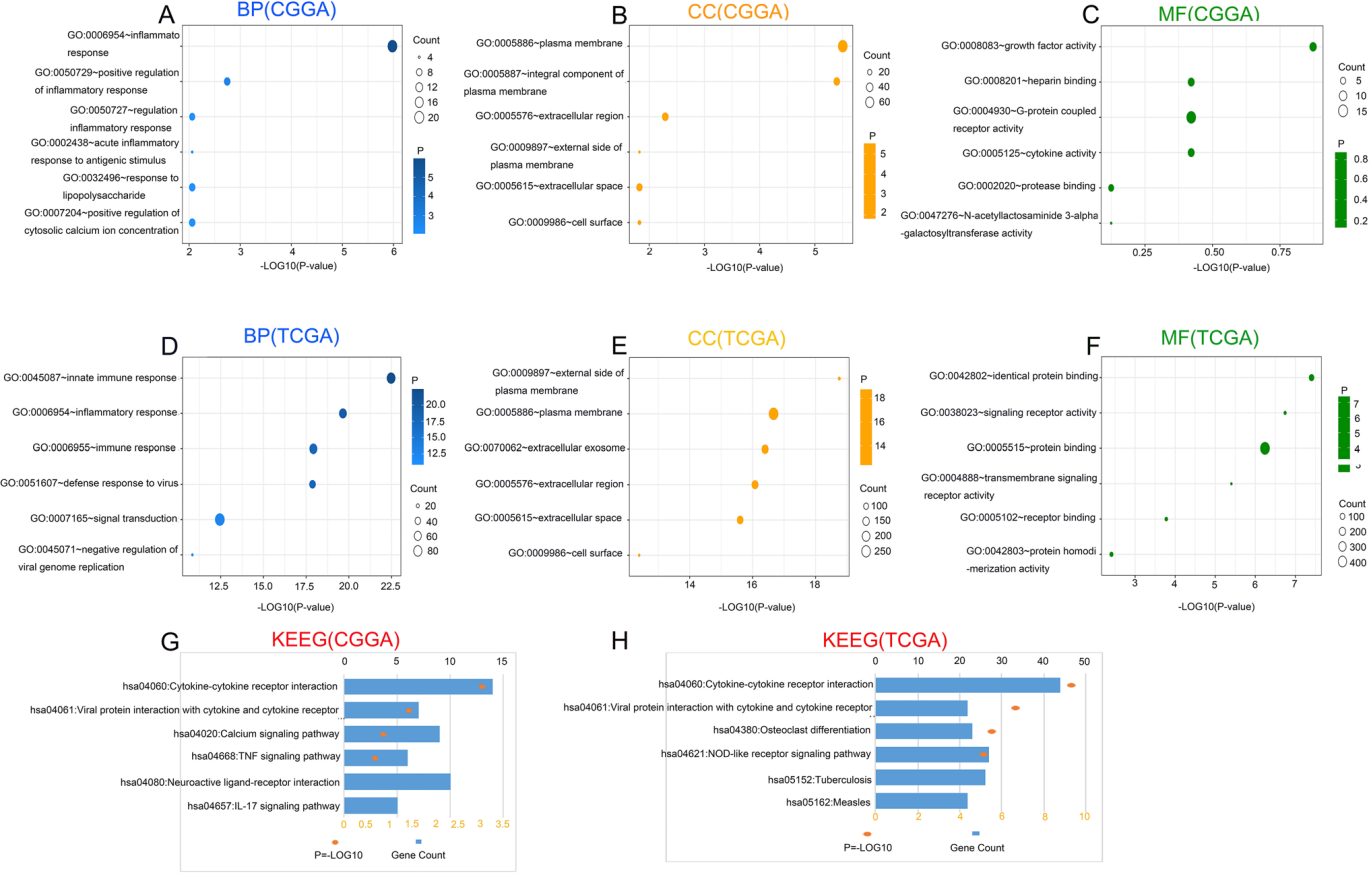
CEACAM1 is closely associated with inflammatory response regulation in gliomas. **A**–**C** Biological processes (BP), cellular components (CC), and molecular functions (MF) are mostly related to CEACAM1 in the Chinese Glioma Genome Atlas (CGGA) database. **D**–**F** Biological processes (BP), cellular components (CC), and molecular functions (MF) are mostly related to CEACAM1 in the TCGA database. **G** Kyoto Encyclopedia of Genes and Genomes (KEGG) pathway analysis of CCEACAM1 in the CGGA database. **H** KEGG pathway analysis of CEACAM1 in the TCGA database

To explore the biological functions related to CEACAM1, the genes most related to CEACAM1 were screened out by Pearson correlation analysis (|R|> 0.05,* P* < 0.05) in the TCGA and CGGA databases. GO and KEGG analysis based on the above gene sets were performed. In the CGGA database, biological processes most related to CEACAM1 include inflammatory response and regulation of inflammatory response (Fig. 8A). Moreover, CEACAM1’s most related cellular components were plasma membrane and integral component of plasma membrane (Fig. 8B). The molecular functions were growth factor activity and heparin binding (Fig. 8C). The most relevant signaling pathway for CEACAM1 is cytokine receptor interaction, including Viral protein interaction with cytokine and cytokine receptor (Fig. 8G). The CEACAM1-related biological processes, cellular components, molecular functions, and signaling pathway in the TCGA database were similar to those in the CGGA database (Fig. 8D–H). These findings suggested that CEACAM1 on glioma cells probably plays an essential role in inflammatory response and regulation of the disease.

### CEACAM1 is an independent prognostic factor for the overall survival of patients with glioma

**Fig. 9:**
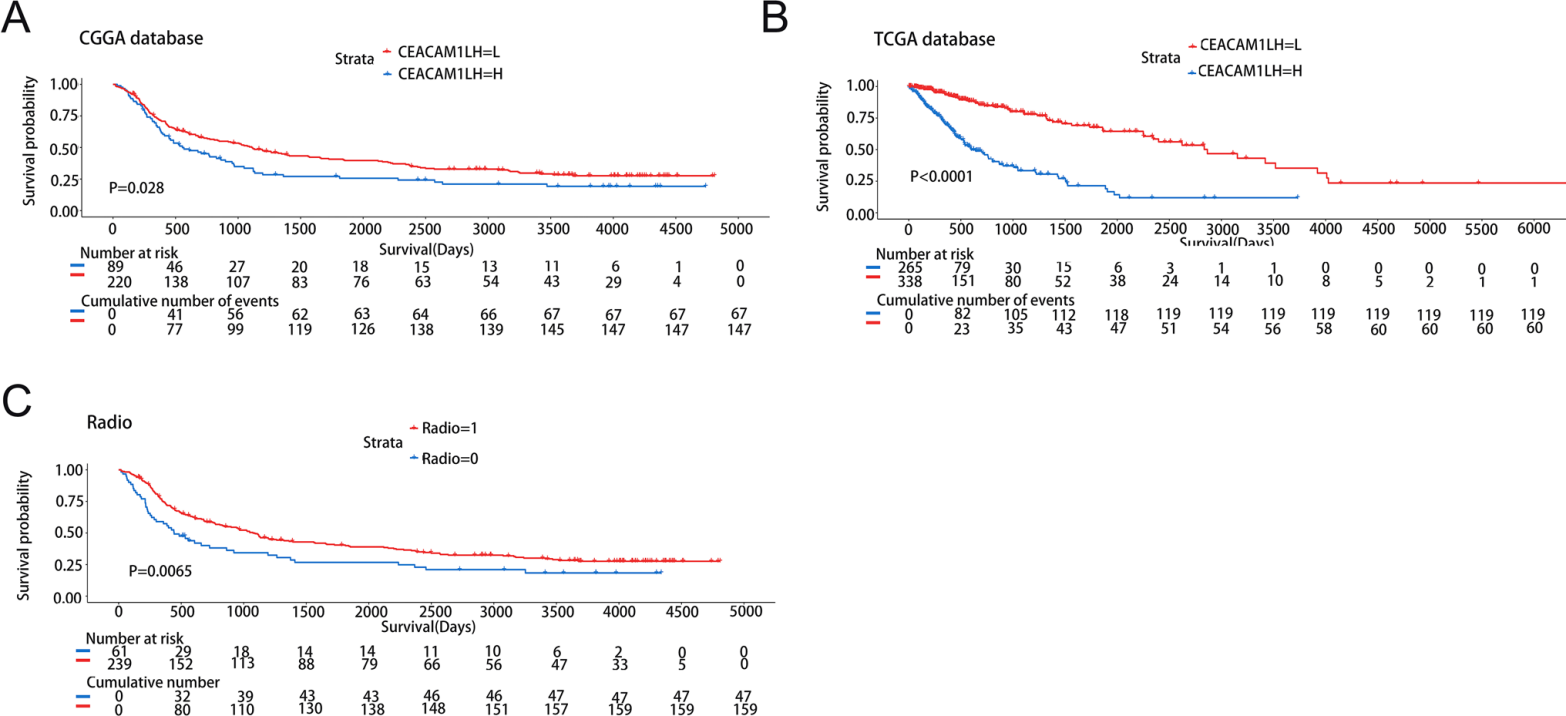
Survival prediction of CEACAM1/Radio in glioma. **A**, **B** Kaplan–Meier analysis of CEACAM1 expression in the Chinese Glioma Genome Atlas (CGGA) and The Cancer Genome Atlas (TCGA) databases. The cutoff of the group is the median expression of CEACAM1.The significance of the prognostic value was tested by a log-rank test. **C** Kaplan­Meier analysis of Radio in the Chinese Glioma Genome Atlas (CGGA) database

To explore the prognostic prediction value of CEACAM1 in patients with glioma, we conducted Kaplan-­Meier and Cox proportional hazard model analyses based on the CGGA and TCGA databases. Patients with higher expression of CEACAM1 had significantly shorter overall survival compared with those with lower CEACAM1 expression in the CGGA database (Fig. 9A). In addition, the prognostic value of CEACAM1 was verified in the TCGA database (Fig. 9B). CEACAM1 expression was a prognostic factor, independent of known prognostic factors, in the Cox regression analysis, including WHO grade, age at diagnosis, IDH mutation, 1p/19q codeletion, and MGMT promoter methylation. These findings revealed that CEACAM1 is an independent prognostic factor in the CGGA (Table 2) and TCGA (Table 3) databases. In addition, we conducted Kaplan–Meier and Cox proportional hazard model analyses based on the CGGA databases to explore the prognostic prediction value of Radio in patients with glioma (TCGA database lacks relevant data). Patients without radio had significantly shorter overall survival compared with those with radio in the CGGA database (Fig. 9C).

**Table 2:** Univariate and multivariate analysis of prognostic parameters in the Chinese Glioma Genome Atlas (CGGA) database overall survival (OS)

Variable	Univariate analysis		Multivariate analysis	
	HR(95%CI)	P-value	HR(95%CI)	P-value
CEACAM1	1.182(1.035–1.348)	0.013	0.796(0.644–0.983)	0.034
WHO grade	5.657(3.917–8.171)	2.48E-20	3.195(2.091–4.884)	0.000
Age	1.614(1.214–2.145)	0.001	1.034(0.746–1.434)	0.842
IDH	0.355(0.269–0.468)	0.355	0.688(0.472–1.001)	0.050
1p/19q	0.170(0.104–0.277)	0.170	0.207(0.123–0.350)	0.000
MGMT	0.830(0.632–1.089)	0.178	0.765(0.644–0.983)	0.089

*CI* Confidence interval; *HR* Hazard ratio; *IDH* Isocitrate dehydrogenase; *WHO* World health organization

**Table 3:** Univariate and multivariate analysis of prognostic parameters in The Cancer Genome Atlas (TCGA) database overall survival (OS)

Variable	Univariate analysis		Multivariate analysis	
	HR(95%CI)	P-value	HR(95%CI)	P-value
CEACAM1	1.679(1.533–1.839)	0.000	1.198(1.050–1.367)	0.007
WHO grade	6.103(3.890–9.574)	0.000	2.721(1.630–4.540)	0.000
Age	5.043(3.348–7.596)	0.000	3.867(2.329–6.420)	0.000
IDH	0.091(0.064–0.129)	0.000	0.268(0.159 – 0.452)	0.000
1p/19q	0.220(0.130 – 0.375)	0.000	0.517(0.278–0.961)	0.037
MGMT	0.312(0.225–0.433)	0.000	0.961(0.650–1.419)	0.840

*CI* Confidence interval; *HR* Hazard ratio; *IDH* Isocitrate dehydrogenase; *WHO* World health organization

## Discussion

Radiotherapy plays an important and irreplaceable role in the treatment of glioma. Previous studies have shown that radiotherapy induces the immunogenic death of tumour cells, the release of large amounts of new tumour-associated antigens (TAAs), and hence the activation of anti-tumour immune responses [7]. Moreover, radiotherapy increases the production of chemokines, promotes the maturation of antigen-presenting cells, and facilitates their infiltration into tumour tissues. Through the above mechanisms, radiotherapy can improve the tumour immune microenvironment, transform “cold” tumour immune landscapes into “hot” ones, and lay the foundation for anti-tumour immunotherapy [8]. Apart from activating immune responses, radiotherapy can also induce the expression of negative immune checkpoint molecules on the surface of tumour cells. For example, radiotherapy promotes the expression of PD-L1 on the surface of lung cancer cells [9]. Preliminary studies have also shown the abnormal expression of the immune checkpoint molecule CEACAM1 in T-cells of patients with glioma, which was closely associated with clinical factors, including the pathological grading of tumours, patient functional status, and the level of cytokines [6].

It is well-known that T-cells constitute the foundation of anti-tumour immune responses, and immune checkpoint molecules can transmit inhibitory signals to T-cells via different pathways, significantly inhibiting the immune response of T-cells [10]. As a transmembrane glycoprotein that belongs to the carcinoembryonic antigen (CEA) family, CEACAM1 has been shown to prevent the activation and proliferation of T-lymphocytes, severely inhibiting their functions [11]. Hence, CEACAM1 blockade enhances the ability of the immune system to kill tumour cells. Previous studies suggested that CEACAM1 serves as a ligand for another important negative immune checkpoint molecule, Tim-3. Interestingly, CEACAM1-Tim3 interactions notably enhance the negative immunoregulatory capacity of Tim-3 and promote tumour evasion of immunosurveillance and immune killing [12].

All these findings have suggested that a combination of radiotherapy with immunotherapy might have a strong synergistic effect in the treatment of glioma. Thus, in this study, we first observed the expression of CEACAM1 on T-lymphocytes in the peripheral blood of patients with glioma before and after radiotherapy and found that the CEACAM1 expression level was significantly elevated after radiotherapy. This finding suggested that radiotherapy might induce a suppressive immune microenvironment in glioma tissues by upregulating the expression of CEACAM1. Subsequently, we utilized C57BL/6 mice and GL-261-Luc cells to construct mouse models of glioma in situ. Such in situ models are syngeneic transplantation models with low risk of immunological rejection, no spontaneous tumour regression, rare extracranial metastases, and high reproducibility; they can also be used to mimic the immune microenvironment in human glioma tissues [13]. Other research indicated that CEACAM1 homophilic interactions restraining multiple effector functions of tumour-infiltrating T-cells [14]. Therefore, we further investigated the therapeutic effect of radiotherapy combined with targeted blockade of CEACAM1 on murine glioma. We found that compared with the control group, the radiotherapy monotherapy, anti-CEACAM1, and combination therapy groups experienced notable inhibition of murine intracranial glioma proliferation and significant extension of murine survival time. Some mice even achieved long-term survival (> 90 d) Fig. 6.

CD8^+^ T cells constitute the most important component of anti-tumour immune responses. Our study suggested the presence of a higher percentage of mouse brain-infiltrating CD8^+^ T-lymphocytes in mice of the combination therapy group than in the control group. Tregs are known to be involved in the negative regulation of immune responses, impairing the anti-tumour immune responses of the body. Studies on glioblastoma suggested a significant increase in the percentage of Tregs in tumour-infiltrating T-cells and in the peripheral blood, with high-grade glioma exhibiting a higher Treg infiltration rate than that of low-grade glioma [15]. The ratio of CD8^+^/Treg was positively correlated with the survival and prognosis of patients, with higher ratios indicating better prognosis [16]. This study showed a decrease in the number of mouse brain-infiltrating Treg cells and an increase in the ratio of CD^+^8/Treg in the combination therapy group, further indicating that a combination of radiotherapy with anti-CEACAM1 treatment can reverse the immunosuppressive microenvironment in glioma tissues, inhibit tumour proliferation, and restore immune responses Fig. 7.

Previous studies have suggested that mice lacking expression of IFN-γ experience an increase in the percentage of myeloid-derived suppressor cells and Tregs, as well as a decrease in the number of tumour-infiltrating CD8^+^ T-cells [17]. Due to the presence of an immunosuppressive microenvironment, effector T-cells often exhibit a state of incapacitation or exhaustion, with the production of IFN-γ serving as an important marker indicating the recovery of the antitumor immunity of CD8^+^ T-cells [18]. In addition, patients with glioma experience elevated levels of an inhibitory cytokine, IL-10, which inhibits the expression of pro-inflammatory factors, mediates the function of Tregs, and inhibits the CD8^+^ T-cell-mediated cytotoxicity and antigen presentation of antigen-presenting cells [19]. Our results showed that the combination therapy group is not better compared to anti-CEACAM1 monotherapy in terms of cytokine release. The reasons may be as follows: (1) Radiation therapy may weaken the immune functions and decrease the level of cytokines [20]. (2) after radiotherapy, the expression of the immune checkpoint molecule CEACAM1 was upregulated, and the CEACAM1 can inhibit the effector functions of TIL (Tumor infiltrates T cells), such as cytotoxicity and IFN-γ release[11]; and the treatment of anti-CEACAM1 can regulate the release of cytokines and activate the immune responses. In addition, our studies revealed higher levels of IFN-γ and lower levels of IL-10 in the peripheral blood of mice in the combination therapy group, indicating that the combination therapy can restore the anti-tumour immune responses of the body by reducing the expression of the IL-10 immunosuppressive factor and restoring the level of IFN-γ Fig. 8.

We observed the proliferation and apoptosis of glioma cells in each group by immunohistochemical staining, and the results showed that the proliferation activity of glioma cells in the combined treatment group decreased, but the apoptosis rate increased significantly. These results suggested that combined radiotherapy and anti-CEACAM1 can induce glioma cells apoptosis and inhibit the proliferative ability of glioma cells, thus can markedly prolong the survival time of the murine intracranial glioma model Fig. 9.

Up to the present, there are only a few comprehensive reports on CEACAM1 in patients with glioma. Using the Chinese Glioma Genome Atlas (CGGA) [21] and The Cancer Genome Atlas (TCGA), we analyzed the CEACAM1 expression profile in different gliomas and further explored the correlation between CEACAM1 expression and clinicopathological characteristics and survival in patients with glioma.

However, there are some limitations in this study. These included its small sample size and not investigating the optimal combination of radiotherapy and immunotherapy by incorporating parameters such as dosage and time window. These issues need to be addressed in future studies.

## Conclusions

The expression of CEACAM1 on T-cells in the peripheral blood of patients with glioma was increased after radiotherapy. In addition, A combination of radiotherapy with anti-CEACAM1 immunotherapy significantly inhibited the proliferation of murine intracranial glioma, extended the survival time of mice, and even restored anti-tumour immunity in some mice.

## Data Availability

The datasets used and/or analysed during the current study are available from the corresponding author on reasonable request.

## Abbreviations

CEACAM1: Carcinoembryonic antigen cell adhesion molecule 1
HE: Haematoxylin and eosin
ELISA: Enzyme linked immunosorbent assay
BIL: Brain infiltrating lymphocytes
IFN-γ: Interferon-γ
IL-10: Interleukin-10
IRS: Immunoreactive scoring
